# Autochthonous Leptospirosis in South-East Austria, 2004–2012

**DOI:** 10.1371/journal.pone.0085974

**Published:** 2014-01-20

**Authors:** Martin Hoenigl, Carina Wallner, Franz Allerberger, Friedrich Schmoll, Katharina Seeber, Jasmin Wagner, Thomas Valentin, Ines Zollner-Schwetz, Holger Flick, Robert Krause

**Affiliations:** 1 Section of Infectious Diseases and Tropical Medicine, Department of Medicine, Medical University of Graz, Graz, Austria; 2 Division of Pulmonology, Department of Medicine, Medical University of Graz, Graz, Austria; 3 Austrian Agency for Health and Food Safety (AGES), Vienna, Austria; Royal Tropical Institute, Netherlands

## Abstract

**Background:**

Leptospirosis is one of the world’s mostly spread zoonoses causing acute fever. Over years, leptospirosis has been reported to occur rarely in Austria and Germany (annual incidence of 0.06/100,000 in Germany). Only imported cases have been on the increase. Objectives of this case-series study were to retrospectively assess epidemiologic and clinical characteristics of leptospirosis illnesses in South-East Austria, to describe risk exposures for autochthonous infections, and to compare patients with imported versus autochthonous infection.

**Methodology/Principal Findings:**

During the 9-year period between 2004 and 2012, 127 adult patients (49 females, 78 males) who tested positive by rapid point-of-care test for *Leptospira*-specific IgM (Leptocheck®) were identified through electronic hospital databases. Follow-up telephone interviews were conducted with 82 patients. A total of 114 (89.8%) of the 127 patients enrolled had acquired leptospirosis within Austria and 13 (10.2%) had potentially imported infections. Most autochthonous cases were diagnosed during the months of June and July, whereas fewest were diagnosed during the winter months. Exposure to rodents, recreational activities in woods or wet areas, gardening, cleaning of basements or huts were the most common risk exposures found in autochthonous infection. Serogroups Australis (n = 23), Sejroe (n = 22), and Icterohaemorrhagiae (n = 11) were identified most frequently by MAT testing in autochthonous infections. Patients with imported leptospirosis were significantly younger, less likely to be icteric and had significantly lower liver transaminase levels (p = 0.004) than those with autochthonous infections.

**Conclusions/Significance:**

Leptospirosis is endemic in South-East Austria. In contrast to reports from other countries we found a relatively high proportion of leptospirosis cases to be female (39% vs. ∼10%), likely the result of differing risk exposures for South-East Austria.

## Introduction

In recent years, leptospirosis has gained increasing attention as an emerging infectious disease of global importance [1]. The clinical manifestations range from asymptomatic infection to severe and potentially fatal illness complicated by septic shock and organ failure [2]–[5]. The broad clinical spectrum is presumably one of the main reasons why diagnosis of this spirochaetal infection remains a challenge for clinicians. Over years, leptospirosis has been reported to occur rarely in Austria and Germany [6], [7]. Diagnostic testing for the infection is therefore performed only in specialized reference hospitals. The low leptospirosis incidence rate reported for Germany and Austria appears discordant, however, with the high rate of *Leptospira* spp. seropositivity among healthy Austrian men identified by Poeppl and colleagues [8].

Hawaii has the highest reported annual incidence rate of leptospirosis in the United States, reaching 1.63 cases per 100,000 inhabitants [9]. Lower annual incidence rates have been reported from Central and Western Europe ranging from 0.06/100,000 per year in Germany over 0.1/100,000 in the United Kingdom and 0.25/100,000 in the Netherlands to 0.5/100,000 in France [6], [7], [10]–[12]. An increasing proportion of the leptospirosis infections diagnosed in many European countries is imported from abroad (25% to 60% of all infections) [10], [12]–[14]. Most of the imported infections have been associated with sporting and adventurous vacation activities abroad [14]–[16].

The objectives of this study were to determine the epidemiology of leptospirosis in South-East Austria, to describe risk exposures for acquiring autochthonous infection, and to compare clinical characteristics of patients with imported to those with autochthonous infection.

## Methods

Over a 9-year period (2004–2012), all patients with clinically compatible illness that have been tested positive by rapid point-of-care (POC) test for *Leptospira*-specific IgM (Leptocheck®, Zephyr Biomedicals, India) at the microbiology laboratory, University Hospital Graz, were included. The hospital has a capacity of more than 1500 beds and serves as reference hospital for about 1 million inhabitants in South-East Austria. If borderline positive test results were obtained with the routine microbiological evaluation, a repeat test was ordered within a few days to confirm the infection.

Microscopic agglutination testing (MAT) was performed on a subset of specimens at the Institute for Veterinary Disease Control in Moedling (AGES), for detection of the causative *Leptospira* serovar. A titre greater than 200 against any of the pathogenic antigens was considered positive. We defined all patients with positive POC test and clinically compatible illness as cases of leptospirosis, independent of MAT result, because the POC IgM test was recently shown to be more sensitive than MAT (85.6% versus 49.8%), with comparable specificity (96.2% versus 98.8%) in a study evaluating results in more than 1500 cases of leptospirosis [17]. In another recent study comparing prospectively three POC tests for *Leptospira*-specific IgM the Leptocheck® IgM POC test, which was also used in this study, showed the best results with an overall sensitivity of 78% and a specificity of 98% [18].

Data regarding course of disease as well as risk exposures (within three weeks before onset of infection) were collected via telephone questionnaires (n = 82; conducted between June 2012 and January 2013) and/or abstracted from electronic hospital databases (n = 127). Infections in patients that had travelled in foreign countries within three weeks before occurrence of symptoms were classified as potentially imported [17]. All other infections were classified as autochthonously acquired.

Questionnaire responses were entered into an electronic database. All statistical analyses were performed using the Statistical Package for Social Sciences version 20.0 (SPSS Inc., Chicago, IL, USA). Continuous data are presented as medians (inter-quartile ranges [IQR]) or means (95% confidence interval [CI]) and categorical data as proportions. Proportions were compared using the chi-squared or Fisher’s exact test as appropriate. Analyses of continuous data were performed using the Mann-Whitney U test or Students T-test as appropriate. Bootstrapping was used to calculate the 95% CI for proportion by gender.

The study was conducted according to the principles expressed in the Declaration of Helsinki and was approved by the local ethics committee, Medical University of Graz. All data presented have been de-identified and are therefore not attributable to individual patients. At our center, information of all patients admitted is automatically stored in the electronic hospital database, and written informed consent of participating patients was waived by the local ethics committee.

## Results

A total of 127 adult patients (range 18–89 years) with positive *Leptospira* specific IgM tests were identified while the test resulted negative in another 794 patients. 114/127 (89.8%) patients had acquired leptospirosis within Austria and 13 (10.2%) had potentially imported infections (Table 1). No outbreak was observed during the study period. In 80/127 (63%) patients single serum samples were received for MAT testing, which turned out positive in 61 (76%) and negative (i.e. titre <200) in 19/80 patients (24%). Cases per year (autochthonous and imported) are shown in Table 1. Median duration of symptoms prior to presentation at the hospital was 5 days (IQR 3–9 days) and the median length of hospitalization was eight days (IQR 5–12 days); neither parameter differed between autochthonous and imported cases. Four autochthonous and two potentially imported cases had severe infections requiring ICU admission secondary to sepsis and organ failures; all six patients survived.

**Table 1:** Demographic data as well as recreational/occupational and residential risk exposures in autochthonous cases (overall, males, females) and imported cases.

Demographic data	AutochthonouscasesN = 114	AutochthonousMalesN = 70	AutochthonousFemalesN = 44	Imported casesN = 13
Male Sex (N; %; 95% CI )	70 (61%; 52–71%)			8
Female Sex (N; %; 95% CI )	44 (39%; 29–47%)			5
Age (years; mean, 95% CI)	33 (26–40)			43 (40–47)
Cases per year				
2004	10			4
2005	16			1
2006	18			3
2007	26			2
2008	23			2
2009	5			1
2010	5			0
2011	5			0
2012	6			0
Risk exposures				
Recreational/Occupational (N; %)				
Activities in woods/wet Areas	44 (39%)	31 (44%)	13 (30%)	7 (54%)
Gardening	36 (32%)	19 (27%)	17 (39%)	2 (15%)
Cleaning up/demolishing basement/hut/attic	28 (25%)	14 (20%)	14 (32%)	2 (15%)
Swimming/snorkelling/diving	9 (8%)	5 (7%)	4 (9%)	5 (38%)
Trekking	4 (4%)	3 (4%)	1 (2%)	5 (38%)
Excavation work	5 (4%)	4 (6%)	1 (2%)	2 (15%)
Camping	1 (1%)	1 (1%)	0	2 (15%)
Channel Digger	3 (3%)	3 (4%)	0	0
Surfing in a river	1 (1%)	1 (2%)	0	1 (8%)
Residential (N; %)				
Exposure to rats/mice	53 (46%)	32 (46%)	21 (48%)	8 (62%)
Contact to cats	29 (25%)	14 (20%)	15 (34%)	1 (8%)
Contact to dogs	20 (18%)	15 (21%)	5 (11%)	3 (23%)
Eating fruits/vegetables from the own garden	19 (17%)	10 (14%)	9 (20%)	0
Pond in surroundings	11 (10%)	9 (13%)	2 (5%)	4 (31%)
Farm Animals	11 (10%)	9 (13%)	2 (5%)	1 (8%)
Food from organic farming- markets ordirectly from farmer	8 (7%)	3 (4%)	5 (11%)	2 (15%)

Abbreviations: 95% CI, 95% confidence intervall; N, number.

### Characterisation of Autochthonous Cases

Overall 114 cases of autochthonous leptospirosis were diagnosed over a 9-year period, which is in average 12.6 cases a year. Considering that the Medical University Hospital Graz serves as a reference hospital for about 1 million inhabitants in South-East Austria this rate would correspond to an autochthonously acquired infection rate of 1.26 per 100,000 inhabitants per year in South-East Austria over the study period. After reaching a peak in 2007–2008 with more than 20 cases reported per year, the number of autochthonous cases declined in 2009 and remained stable at 5 to 6 cases per year through 2012 (Table 1). The male to female ratio was 1.6∶1 for autochthonous cases. Most autochthonous cases were diagnosed during the months of June and July (n = 16 each), followed by October (n = 13), August (n = 12), April (n = 11) and March (n = 10), while fewer infections were diagnosed during the winter months (4 in December, 6 in January and 3 in February).

Commonly reported exposures for acquiring leptospirosis were activities in woods and wet areas, and exposure to rodents. Gardening or eating fruits/vegetables from the own garden/organic-farming markets was reported by 31/44 (70.5%) of female autochthonous cases. The proportion of patients reporting various risk exposures by subgroup are depicted in Table 1.

Serum samples were tested by MAT for 72/114 (63%) patients with autochthonous infection and 54 (75%) were positive. The following serogroups were identified: Australis (n = 23), Sejroe (n = 22), Icterohaemorrhagiae (n = 11), Ballum (n = 10), Grippotyphosa (n = 7), Canicola (n = 4), Bataviae (n = 2), Pyrogenes and Hebdomadis (n = 1).

The most frequently reported symptoms include fever (80/114; 70%), myalgia and/or arthralgia (41/114; 36%), abdominal pain and/or diarrhea (34/114; 30%), general weakness (31/114; 27%), jaundice (31/114; 27%), headache (24/114; 21%) and nausea and/or sickness (20/114; 18%). Laboratory results at admission revealed that 44/114 patients (39%) had thrombocytopenia (<140.000 cells/µL; 25/44 even <100.000 cells/µL). Elevated alanine aminotranferase levels (ALT >2 times normal value) were found in 55/114 (48%). There was no significant difference in the median ALT level for patients aged <50 years compared to older patients (median 75, IQR 29–456 vs median 80, IQR 42–149, respectively). Elevated serum creatinine levels (>1.2 mg/dL) were found in 45 (39%) patients with 25 patients having serum creatinine levels >2 mg/dL.

### Comparison between Autochthonous and Imported Infections

Of the 13 cases with imported infections, five had been in South-East Asia within the incubation period, two in Africa, one in South America and five in Central Europe. Serogroups for the imported cases (samples received for MAT testing in 8/13 patients; positive result in 7/8) included Australis (n = 3), Ballum, Grippotyphosa, Icterohemorrhagiae (each n = 2), and Bataviae, Canicola, and Sejroe in one case each.

Patients with imported leptospirosis were significantly younger than those with autochthonous infections (p = 0.045; Students T Test). Jaundice at presentation was less common (p = 0.037; Fishers exact test) and ALT levels were significantly lower in patients with imported leptospirosis compared to autochthonous infections (median 24, IQR 14–40 U/L versus median 76, IQR 30–292; p = 0.004; Mann Whitney U test). No significant differences were found for other parameters.

## Discussion

We found that leptospirosis is endemic in South-East Austria. Our findings would correspond to an autochthonously acquired infection rate of 1.26 per 100,000 inhabitants per year in South-East Austria over the study period. The estimated incidence probably reflects the more severe end of the clinical spectrum for leptospirosis, as mild forms of this disease are more likely to remain unrecognized and may have presented to smaller peripheral hospitals or family doctors, i.e. settings where testing for leptospirosis is yet not performed.

Therefore we believe that the actual rate of leptospirosis in South-East Austria may be much higher than the rate reported here. Although it is required by law to report basic demographic data of all serologically confirmed leptospirosis cases to the Austrian Government the official numbers may suffer from underreporting for two reasons: (i) the fact that leptospirosis cases are rarely confirmed by serological tests in Austria as diagnostic testing for the infection is performed in a few specialized reference hospitals only, (ii) the suboptimal reporting behaviour of clinicians. It is interesting that a recent cross-sectional study demonstrated serological evidence of a high rate of exposure to *Leptospira* spp. among Austrian males [8].

In accordance with the literature exposure to rats or mice has been present in almost 50% of cases of our study cohort [10]. Other important risk exposures included recreational activities in woods or wet areas, gardening as well as cleaning basements or huts. Imported cases were less often icteric and presented with significantly lower transaminase levels when compared to autochthonous cases. This finding is consistent with a previous study of 60 cases of leptospirosis mostly from Germany [2]. In contrast to that study and other reports, we found, however, that in our hospital the number of both imported and autochthonous cases decreased over the study period [2], [10]. While changes in temperature and rainfall may have been contributing factors, the reason for the decrease in autochthonous infections from 2008 to 2009 remains unknown [19].

Another surprising finding was that in our study 39% (95% CI 29–47%) of autochthonous leptospirosis cases occurred in females which stands in contrast to data from Hawaii, the Netherlands and UK where females accounted for less than 10% of leptospirosis cases [9], [10], [12]. In our setting the most frequently identified serogroups were Australis, Sejroe and Icterohaemorrhagiae. In accordance with a previous study from Austria and in contrast to the Netherlands, where the serogroup Canicola had disappeared after 1966, we found four patients autochthonously infected by this serogroup [10]. Two of these four patients had reported contact to dogs prior to occurrence of symptoms.

### Limitations of the Study

A number of possible limitations have to be taken into account when interpreting the results of this study. First, we did not use the standard case definition that relies mainly on MAT testing but instead defined all patients with positive POC test and clinically compatible illness as cases of leptospirosis, independent of MAT result. We believe this approach is justifiable because the POC IgM test was recently shown to be more sensitive than MAT and has comparable specificity. Nevertheless, comparisons with other studies may be difficult due to the differences in the case definitions used. Second, the study design was retrospective and did not include a control group; therefore we cannot implicate specific risk exposures as the likely source of leptospirosis infection. Also, recall regarding exposures might have been adversely affected or biased by the fact that, in many cases, the phone interviews occurred years after the illness. Lastly, the overall sample size was relatively small and this limited our ability of making statistical comparisons between subsets of cases.

### Conclusions

In summary, we report a high rate of leptospirosis occurring in South-East Austria between 2004 and 2012. The vast majority of cases were autochthonously acquired. The main risk exposures for acquiring leptospirosis reported were activities in woods and wet areas as well as exposure to rodents.

## References

[1] Vijayachari P, Sugunan AP, Shriram AN (2008) Leptospirosis: An emerging global public health problem. J Biosci 33: 557–569. 19208981 10.1007/s12038-008-0074-z

[2] Hoffmeister B, Peyerl-Hoffmann G, Pischke S, Zollner-Schwetz I, Krause R, et al (2010) Differences in clinical manifestations of imported versus autochthonous leptospirosis in austria and germany. Am J Trop Med Hyg 83: 326–335. 20682876 10.4269/ajtmh.2010.10-0040 PMC2911179

[3] Levett PN (2001) Leptospirosis. Clin Microbiol Rev 14: 296–326. 11292640 10.1128/CMR.14.2.296-326.2001 PMC88975

[4] Spichler A, Athanazio D, Buzzar M, Castro B, Chapolla E, et al (2007) Using death certificate reports to find severe leptospirosis cases, brazil. Emerg Infect Dis 13: 1559–1561. 18258007 10.3201/eid1310.070150 PMC2851519

[5] Tubiana S, Mikulski M, Becam J, Lacassin F, Lefevre P, et al (2013) Risk factors and predictors of severe leptospirosis in new caledonia. PLoS Negl Trop Dis 7: e1991. 23326614 10.1371/journal.pntd.0001991 PMC3542117

[6] Jansen A, Schoneberg I, Frank C, Alpers K, Schneider T, et al (2005) Leptospirosis in germany, 1962–2003. Emerg Infect Dis 11: 1048–1054. 16022779 10.3201/eid1107.041172 PMC3371786

[7] Radl C, Muller M, Revilla-Fernandez S, Karner-Zuser S, de Martin A, et al (2011) Outbreak of leptospirosis among triathlon participants in langau, austria, 2010. Wien Klin Wochenschr 123: 751–755. 22105111 10.1007/s00508-011-0100-2

[8] Poeppl W, Orola M, Herkner H, Muller M, Tobudic S, et al (2013) High prevalence of antibodies against leptospira spp. in male austrian adults: A cross-sectional survey, april to june 2009. Euro Surveill 18: 20509. 23806296 10.2807/1560-7917.es2013.18.25.20509

[9] Katz AR, Buchholz AE, Hinson K, Park SY, Effler PV (2011) Leptospirosis in hawaii, USA, 1999–2008. Emerg Infect Dis 17: 221–226. 21291592 10.3201/eid1702.101109 PMC3204774

[10] Goris MG, Boer KR, Duarte TA, Kliffen SJ, Hartskeerl RA (2013) Human leptospirosis trends, the netherlands, 1925–2008. Emerg Infect Dis 19: 371–378. 23622144 10.3201/eid1903.111260 PMC3647640

[11] Baranton G, Postic D (2006) Trends in leptospirosis epidemiology in france. sixty-six years of passive serological surveillance from 1920 to 2003. Int J Infect Dis 10: 162–170. 16298537 10.1016/j.ijid.2005.02.010

[12] Forbes AE, Zochowski WJ, Dubrey SW, Sivaprakasam V (2012) Leptospirosis and weil’s disease in the UK. QJM 105: 1151–1162. 22843698 10.1093/qjmed/hcs145

[13] Perra A, Servas V, Terrier G, Postic D, Baranton G, et al (2002) Clustered cases of leptospirosis in rochefort, france, june 2001. Euro Surveill 7: 131–136. 12631991 10.2807/esm.07.10.00361-en

[14] Picardeau M (2013) Diagnosis and epidemiology of leptospirosis. Med Mal Infect 43: 1–9. 23337900 10.1016/j.medmal.2012.11.005

[15] Sejvar J, Bancroft E, Winthrop K, Bettinger J, Bajani M, et al (2003) Leptospirosis in “eco-challenge” athletes, malaysian borneo, 2000. Emerg Infect Dis 9: 702–707. 12781010 10.3201/eid0906.020751 PMC3000150

[16] Lagi F, Corti G, Meli M, Pinto A, Bartoloni A (2013) Leptospirosis acquired by tourists in venice, italy. J Travel Med 20: 128–130. 23464722 10.1111/j.1708-8305.2012.00669.x

[17] Limmathurotsakul D, Turner EL, Wuthiekanun V, Thaipadungpanit J, Suputtamongkol Y, et al (2012) Fool’s gold: Why imperfect reference tests are undermining the evaluation of novel diagnostics: A reevaluation of 5 diagnostic tests for leptospirosis. Clin Infect Dis 55: 322–331. 22523263 10.1093/cid/cis403 PMC3393707

[18] Goris MG, Leeflang MM, Loden M, Wagenaar JF, Klatser PR, et al (2013) Prospective evaluation of three rapid diagnostic tests for diagnosis of human leptospirosis. PLoS Negl Trop Dis 7: e2290. 23875034 10.1371/journal.pntd.0002290 PMC3708816

[19] [Anonymous]. Jahrbuch: Klimaübersicht österreich.

